# The benefits of an integrated liaison psychiatry and dermatology service for complex dermatology Patients—a case series

**DOI:** 10.1002/ski2.159

**Published:** 2022-10-05

**Authors:** Victoria Roberts, Jonathan Chan, Jo Davies, Janet Angus

**Affiliations:** ^1^ University Hospitals Bristol NHS Trust Bristol UK; ^2^ Avon and Wiltshire Partnership Bath UK; ^3^ University of Bristol Bristol UK

## Abstract

Psychodermatology is a specialist area which refers to the assessment and treatment of the psychosocial aspects of dermatology. This includes the management of patients with primary psychiatric disorders, psychosocial co‐morbidities of existing skin disease or psychological distress caused by their skin conditions. We report the benefits and cost savings of a recent pilot of an integrated service of a consultant dermatologist and a liaison psychiatrist providing coordinated care to complex psychodermatology patients.

## Graphical abstract

Psychodermatology refers to the management of psychosocial aspects of dermatology. We report the benefits and cost savings of an integrated service of a dermatologist and a liaison psychiatrist providing coordinated care to complex patients.

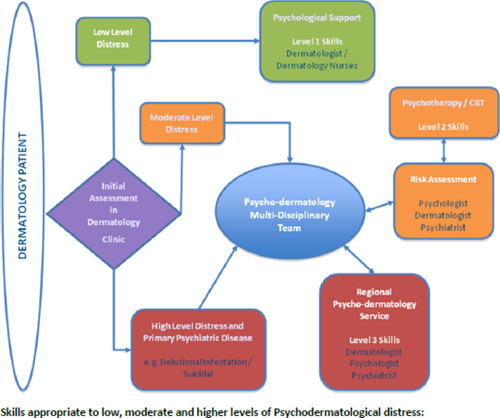

1

**What's already known about this topic?**

- Patient benefit and cost‐effectiveness from an integrated service has been demonstrated by reports from other clinics with joint dermatology and psychology provisions.

**What does this report add?**

- This report provides further evidence for the need, cost‐effectiveness and impact on quality outcomes of the addition of psychiatric input in the care of complex dermatology patients. Our work particularly highlights the benefits of specialist diagnostic and management input of the liaison psychiatrist and how this improves engagement, assessment and cost‐effectiveness of care.

## 2 INTRODUCTION

Psychodermatology is an important consideration in dermatological conditions. The subspeciality focuses on the interface of skin presentations and mental health. The categories of presentations have been described by Koo and Lee.^1^

The British Association of Dermatologists (BAD) Working Party report (2012) noted that 85% of dermatology patients reported the psychosocial impact of their skin disease to be significant and 12.7% had experienced suicidal ideation (8% in controls).^2^

National Institute of Clinical Excellence (NICE) has recognised the importance of psychological approaches.^3^, ^4^, ^5^ This patient group often consults widely and can become high impact users of primary care, dermatology and other acute specialties.

It has been demonstrated by research summarised in the All‐Party Parliamentary Group on Skin 2020 report that multidisciplinary (MDT) psychodermatology clinics offer significant cost effectiveness compared to managing these patients within generalist healthcare settings.^6^

## 3 SERVICE OVERVIEW

**FIGURE 1:**
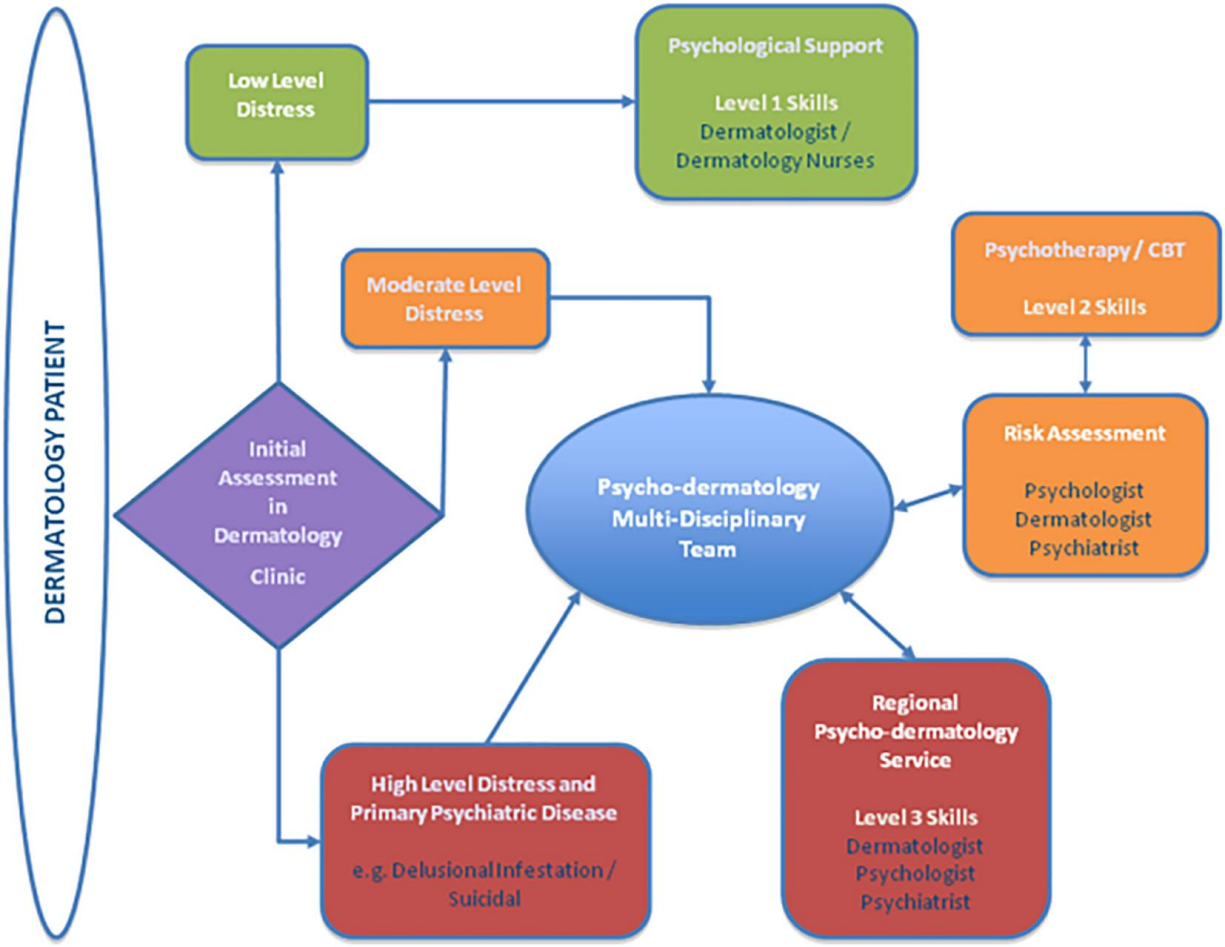
Stepped provision of psychodermatology services^2^

Despite consensus for the need of psychodermatology provision, services are still limited. Our service operates at a supra‐regional level, serving the Southwest and South Wales. The model of care was a stepped care service as outlined in Figure 1.

A senior liaison psychiatry registrar with an interest in psychodermatology worked within the clinic supervised by a consultant psychiatrist. A funding application has been sent for her to remain in post after final consultant accreditation.

**FIGURE 2:**
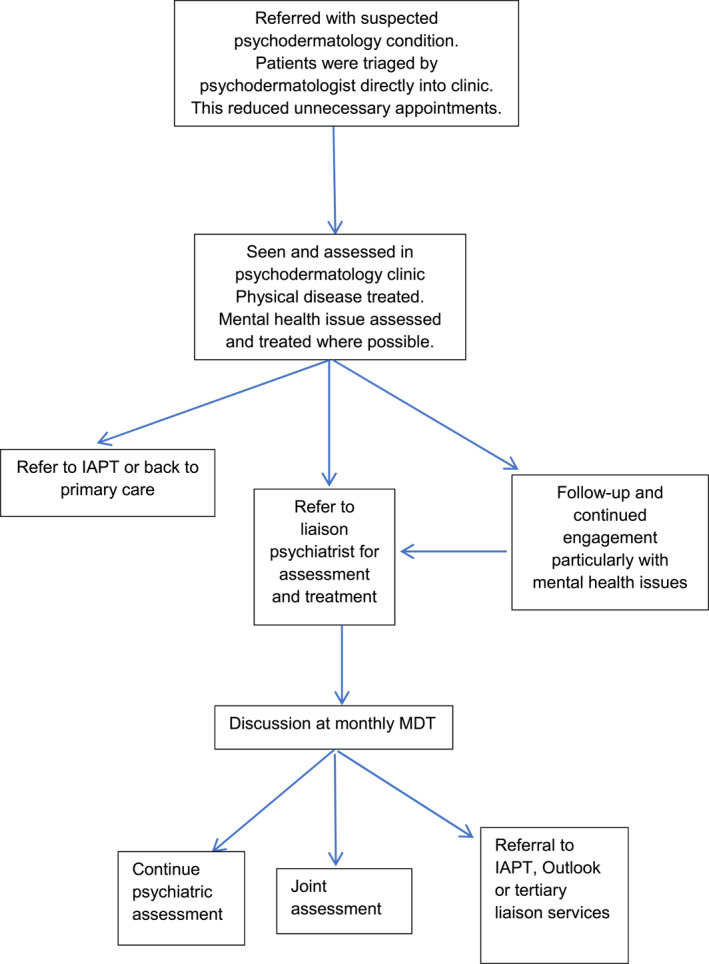
Structure and referral process of psychodermatology service

The clinic was structured and run as outlined in Figure 2. The dermatologist has clinics three times a month. The psychiatrist did two clinics a week. The average waiting time was 3 months.

In the 6 months between July 2021–January 2022, 57 patients were assessed in the dermatologist's clinic. See Table 1 for demographics of the patients referred to the psychodermatology service.

**TABLE 1:** Demographics of patients referred to psychodermatology service

Demographic	Number (%)
Gender	
Female	34 (60)
Male	23 (40)
Average age	40 years
Disease	
Dermatology disease (e.g., acne, HS, eczema) and severe mental health issue	19 (33)
Medically unexplained symptoms	8 (14)
Compulsive skin picking	9 (16)
Body dysmorphic disorder	6 (11)
Delusion of infestation/psychotic illness	12 (21)
Dermatitis artefacta	3 (5)

Thirty three patients were referred to the psychiatrist. See Table 2 for demographics and conditions of patients referred.

**TABLE 2:** Demographics of patients referred to liaison psychiatrist

Demographic	Number (%)
Gender	
Female	22 (67)
Male	11 (33)
Average age	39
Disease	
Dermatology disease (e.g., acne, HS, eczema) and severe mental health issue	10 (30)
Medically unexplained symptoms	3 (9)
Compulsive skin picking	6 (18)
Body dysmorphic disorder	4 (12)
Average number of appointments required	3
Did not attend	3 (9)

There was significant need for psychiatric input for psychodermatology patients. Several had complex presentations due to multiple comorbidities. Scoring (GAD7 and PHQ9) suggested moderate to severe disease burden in these patients. 88% required treatment with psychiatric medication. 40% were deemed inappropriate for interventions in primary care (Improving Access to Psychological Therapy [IAPT]) but didn't meet the threshold for secondary care.

## 4 CASE SERIES

Below are six patient examples who attended clinic for assessment by the dermatologist. Five (patients B–F) were reviewed by a psychiatrist. By attending the clinic, they were able to access secondary mental health services which they wouldn't have been able to access via the GP as they didn't meet the severity threshold.

Patient A presented with severe eczema, compulsive skin picking and recurrent infections. This was so severe that it affected her ability to mobilise. She had post‐traumatic stress disorder following overuse of steroids leading to red skin syndrome. She had a history of obsessive‐compulsive disorder (OCD) and generalised anxiety (GAD) with episodes of suicidal ideation. She had previous contact with mental health services and IAPT. The cyclical nature of her symptoms was identified and a diagnosis of premenstrual dysphoric disorder (PMDD) was made. This was confirmed by gynaecology and she started GnRH analogues. This referral altered the treatment and management of her condition. With support from the clinic she was able to start topical steroids to manage her eczema.

Patient B presented with lifelong severe eczema which was managed in secondary care since childhood and attended eight specialist units across the UK. He was inconsistently adherent to systemic and topical medication resulting in poor disease control and recurrent attendances to acute services. He was assessed in our service and received a diagnosis of bipolar disorder in conjunction with his atopic dermatitis. The patient elected to have psychotherapy rather than take psychotropic medication.

Patient C presented with a crawling sensation over her scalp. She felt she was infested with mites which impacted significantly on her quality of life. She had a past diagnosis of anxiety and moderate depression. She applied various erosive topical preparations to her scalp. Following our assessment, she was started on low dose risperidone and her symptoms resolved. However, she subsequently developed a sudden deterioration in her mental state following a corticosteroid injection for arthritis. A severe depressive disorder with psychomotor agitation was diagnosed. She was commenced on mirtazapine in addition to increased doses of risperidone. She improved and has now accessed IAPT.

Patient D presented with episodic skin difficulties including a burning sensation, pain, redness and swelling which was often precipitated by increased body temperature caused by hot environments. She struggled with temperature regulation which impacted significantly on her quality of life. She had a previous psychiatric history which included depression, suicidal ideation and bulimia. Subsequently, she had been diagnosed with Autism Spectrum Disorder (ASD) and Attention Deficit Hyperactivity Disorder (ADHD). In our clinic, she was diagnosed with Erythromelalgia as a cause for her skin symptoms, which was possibly also compounded by sensory processing difficulties related to her ASD. She was also diagnosed with GAD. She had difficulty tolerating previous psychotropic medications. She was trialled on pregabalin for anxiety which she tolerated and this appeared to improve her anxiety and skin symptoms.

Patient E presented with a long history of compulsive skin picking. This was associated with a significant anxiety disorder. She had been prescribed a Selective Serotonin Reuptake Inhibitor and had seen IAPT. In our services, she was diagnosed with OCD and GAD. Her skin picking disorder was utilised as a coping strategy to manage the tension associated with her symptoms and was prescribed sertraline. She engaged with psychotherapy services and had significant improvement in her symptoms. She was discharged from our service.

Patient F presented with sensation of her hair twisting and coiling under her scalp which she felt was affecting other parts of her body. This was associated with intense dysaesthesia and an overwhelming sensation that she was being strangled by her hair. She had a past history of anxiety, depression, complex trauma and craniofacial pain syndrome. Her beliefs regarding her scalp and hair increased in their intensity and were deemed to be delusional. Initially she was commenced on risperidone and began to develop some insight. Unfortunately, she then experienced a significant deterioration in her mood and became increasing suicidal. She was supported by the Crisis team. We worked in conjunction with them and changed her antipsychotic medication to olanzapine and duloxetine. Her mood and appetite have steadily improved. She has gained further insight into her symptoms and has accessed cognitive behavioural therapy (CBT) for psychosis.

All patients were discussed with the MDT. See Table 3 for outcomes.

**TABLE 3:** MDT involvement and outcome

Patient	MDT discussion
Patient A	This patient benefited from a broader assessment of her clinical issues and by identifying the cyclical nature of her symptoms. In conjunction with a liaison psychiatrist and gynaecologist, we were able to correctly diagnose her with PMDD. This significantly affected her treatment regime.
Patient B	The multi‐disciplinary working highlighted and then confirmed the diagnosis of bipolar disorder. In conjunction with the psychotherapeutic consultations, the patient was able to understand that he was inadvertently non‐compliant with eczema medication because he associated the symptoms of an eczema flare with a manic state for example, lack of sleep and agitation. He erroneously felt that this was associated with productivity. Understanding his reasons for non‐compliance has allowed him to be discharged from dermatology and now manages his eczema successfully with topical treatments.
Patient C	The multidisciplinary working was particularly helpful in managing her acute mental health deterioration. The patient benefited from early additional treatment from her sudden depressive disorder and was managed very successfully with an escalation of psychiatric care in a crisis phase.
Patient D	The integrated approach was successful in managing this complex physical presentation as it was important for the patient to have their physical symptoms understood and investigated. She is very intolerant of medications so we have had to work closely not only within the psychodermatology service but also with the ASD specialist service.
Patient E	This patient was successfully and efficiently treated with the correct treatment of the major anxiety disorder causing the compulsive skin picking presentation. This highlights the benefits both for the patient in correctly managing their disease and the efficient use of healthcare resources.
Patient F	The integration in care was essential for this patient who presented to the dermatologist with a significant psychotic illness. Without an integrated medical and mental health model we don't believe this patient would have engaged so successfully with assessment and treatment.

Abbreviations: ASD, Autism Spectrum Disorder; MDT, multidisciplinary; PMDD, premenstrual dysphoric disorder.

## 5 COST‐EFFECTIVENESS OF SERVICE

A cost analysis of pre‐ and post‐psychodermatology clinic for six patients was conducted and is summarised in Table 4. This was calculated using patient health records to determine the number of presentations to healthcare providers before and after accessing the clinic. This was cross‐checked with patients when health records weren't available. Cost for GP appointments were estimated at £30. Hospital attendances and investigations were calculated using national tariffs.^7^ When there was significant use of systemic therapy, this was also included into the cost analysis using tariffs from the British National Formulary.

**TABLE 4:** Estimated costs in six patients attending the psychodermatology clinic

Patient	A	B	C	D	E	F
Diagnosis	Eczema + dermatillomania + PMDD	Eczema + bipolar disorder	Sensory disturbance + severe depression with psychomotor agitation	Somatic symptoms + ADHD + ASD	Compulsive skin picking and OCD	Complex trauma, craniofacial pain syndrome, delusional disorder
Cost before attending clinic (£)	1238	8857	533	2134	525	1131
Cost after attending clinic (£)	262	942	209	357	135	283
Savings (£)	976	7915	324	1777	390	848
Average saving per patient = £2038.33						

Abbreviations: ADHD, Attention Deficit Hyperactivity Disorder; ASD, Autism Spectrum Disorder; OSD, obsessive‐compulsive disorder; PMDD, premenstrual dysphoric disorder.

## 6 PATIENT FEEDBACK

**FIGURE 3:**
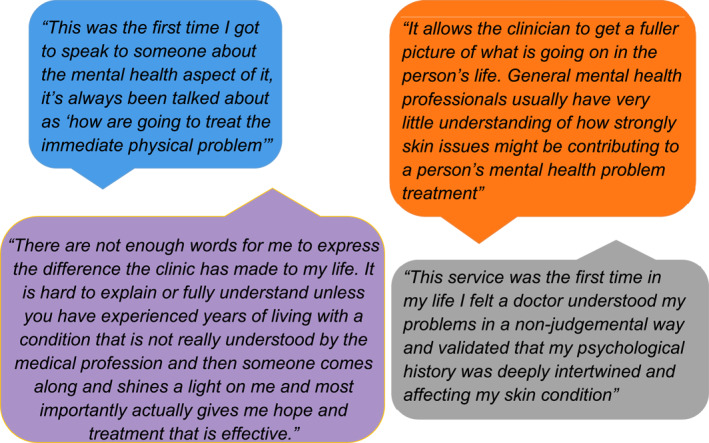
Patient responses to questionnaire rating the service

A survey of patients suggested that 88% felt listened to at the clinic and qualitative feedback demonstrates patients valued our service. See Figure 3 for freetext comments.

## 7 DISCUSSION

Liaison psychiatry input was vital in facilitating the correct diagnosis and management of the mental health issues presenting to our clinic, especially in examples where mental state significantly deteriorated. Expertise in instigating early proactive management of acute deterioration was invaluable. Co‐ordination of care and liaison with other mental health professionals was vital.

The Royal College of Psychiatrists Faculty of Liaison report (2013) suggests that mental health problems occur in 30%–60% of hospital outpatients.^8^ The extra cost of physical healthcare in general hospitals associated with co‐morbid mental health problems is approximately 15% of annual expenditure.^8^

Despite the common occurrence of mental health issues in hospital's outpatients, general medical physicians have no training in the assessment or management of psychiatry disorders post qualification. This results in psychiatric diagnosis being missed and inappropriately managed.

The dermatology curriculum requires dermatologists to receive training in psychodermatology. In this pilot, trainees received training alongside the psychiatrist. This resulted in improved understanding and ability to manage these patients more effectively and initiate appropriate medication.

Our review supports the recent ‘Getting it Right First Time’ dermatology report recommending improved access and expansion in psychodermatology services. They recognised the quality and cost‐effectiveness in reduction of misdiagnosis, duplication and unnecessary investigation that this model of integration of services provides.^9^

Our data supports the evidence collated nationally suggesting significant cost‐savings to the regional health economy through utilising a multidisciplinary psychodermatology clinic. Patients of this type otherwise consult widely across primary and secondary care, having multiple unnecessary investigations, and failing to receive the correct diagnosis or treatment.

All patients featured in the series have consented to their stories being published.

## AUTHOR CONTRIBUTIONS

**Victoria Roberts**: conceptualisation; formal analysis; investigation; methodology; project administration; visualisation; writing – original; writing review and editing. **Jonathan Chan**: conceptualisation; formal analysis; investigation; methodology; project administration; visualisation; writing – review and editing. **Jo Davies**: conceptualisation; funding acquisition; investigation; resources; supervision; writing – review and editing. **Janet Angus**: conceptualisation; investigation; resources; supervision (lead); visualisation; writing – review and editing.

## CONFLICT OF INTEREST

None to declare.

## ETHICS STATEMENT

Not applicable.

## Data Availability

Research data are not shared.
